# Prefrontal Computation as Active Inference

**DOI:** 10.1093/cercor/bhz118

**Published:** 2019-07-10

**Authors:** Thomas Parr, Rajeev Vijay Rikhye, Michael M Halassa, Karl J Friston

**Affiliations:** 1 Wellcome Centre for Human Neuroimaging, Institute of Neurology, University College London, WC1N 3BG, UK; 2 Department of Brain and Cognitive Science, Massachusetts Institute of Technology, Cambridge, MA 02139, USA; 3 McGovern Institute for Brain Research, Massachusetts Institute of Technology, Cambridge, MA 02139, USA; 4 Stanley Center for Psychiatric Genetics, Broad Institute, Cambridge, MA 02139, USA

**Keywords:** active inference, attention, decision-making, prefrontal cortex, working memory

## Abstract

The prefrontal cortex is vital for a range of cognitive processes, including working memory, attention, and decision-making. Notably, its absence impairs the performance of tasks requiring the maintenance of information through a delay period. In this paper, we formulate a rodent task—which requires maintenance of delay-period activity—as a Markov decision process and treat optimal task performance as an (active) inference problem. We simulate the behavior of a Bayes optimal mouse presented with 1 of 2 cues that instructs the selection of concurrent visual and auditory targets on a trial-by-trial basis. Formulating inference as message passing, we reproduce features of neuronal coupling within and between prefrontal regions engaged by this task. We focus on the micro-circuitry that underwrites delay-period activity and relate it to functional specialization within the prefrontal cortex in primates. Finally, we simulate the electrophysiological correlates of inference and demonstrate the consequences of lesions to each part of our in silico prefrontal cortex. In brief, this formulation suggests that recurrent excitatory connections—which support persistent neuronal activity—encode beliefs about transition probabilities over time. We argue that attentional modulation can be understood as the contextualization of sensory input by these persistent beliefs.

## Introduction

Beyond classical reflexes, most interesting behaviors rely upon the use of past information to plan future actions. This implies a temporal discrepancy between a sensation and the action informed by that sensation (Fuster 1990; Coull et al. 2010). For example, during scene construction, visual data garnered from previous fixations inform where we will look next (Mirza et al. 2016; Parr and Friston 2017a). In conversation, our interpretation of, and response to, the last word in a sentence depends upon the first (Montgomery 1995). Imitation involves viewing before replicating another’s movements (Mary et al. 1988). The prefrontal cortex appears to be crucial for solving problems that involve temporal dependencies of this type. Early neuropsychological works (Ferrier 1886; Luria 1980; Szczepanski and Knight 2014), including primate lesion studies (Jacobsen 1935; Harlow et al. 1952), suggest a deficit in “immediate recall” following damage to frontal lobe areas. Subsequently, it has been shown that prefrontal regions are vital for the performance of “delay-period” working memory tasks (Fuster and Alexander 1970; Passingham 1985; Owen et al. 1990) and that these regions house cells that exhibit persistent activity during delays (Fuster 1973; Funahashi et al. 1989). Furthermore, accounts of the role of the (medial) prefrontal cortex in emotional decision-making call upon future (interoceptive) sensory consequences of a decision (Damasio 1996). These all imply an important role for the prefrontal cortex in making inferences based upon the past and the future.

To attempt to understand the computational architecture required for temporally deep behaviors, we derive a Bayes optimal solution to a delay-period task that has recently been used in rodent studies (Wimmer et al. 2015; Schmitt et al. 2017) and is closely analogous to tasks used for human research (Barceló et al. 2000; Griffin and Nobre 2003; Lepsien and Nobre 2007; Astle et al. 2009; Kuo et al. 2009; Lepsien et al. 2011). The task calls upon inferences about an attentional context, based upon a cue. This information must be maintained through a delay period and then used to inform a behavioral outcome. Successful performance of the task requires a capacity to contextualize new information based on what has previously been observed and to use past information when making a decision. The prefrontal cortex is uniquely placed to coordinate this task, due to its dense connectivity with a range of brain areas. This property, sometimes described in terms of “rich-club hubs” (van den Heuvel and Sporns 2011), facilitates the context-sensitive (cognitively flexible) inferences that must be performed during this task.

In this study, we treat this task as an inference problem and describe the computational machinery that a Bayes optimal agent might use to solve this problem. Our aim is to relate these inferential processes to the neuronal architectures found in the prefrontal cortex and to the connections between regions of the frontal cortices. In the following, we start with an overview of active inference. We then describe the form of the generative model required for this task and of the message passing (Winn and Bishop 2005; Dauwels 2007) it entails. Finally, we simulate the electrophysiological correlates of the implicit belief updating (Friston et al. 2017a) in distinct cell populations in a synthetic prefrontal cortex—and show the effects of simulated lesions on this updating.

In short, this paper tries to establish the construct validity of Bayesian belief updating—in the setting of deep temporal models—in relation to empirical electrophysiology (e.g., delay-period activity) and neuropsychology (e.g., damage to the lateral prefrontal cortex). In subsequent work, we will use this scheme to characterize interactions between the prefrontal cortex and thalamus based upon behavior and in vivo electrophysiology, using the same experimental paradigm simulated below.

## Active Inference

Active inference is a formal approach to describing optimal behavior (Friston et al. 2010) that derives from the need for animals to engage in “self-evidencing” behavior (Hohwy 2016). This imperative becomes obvious when we consider a concrete example. For a person to exist, their temperature must remain around 37°C. As such, a measurement (by a thermoreceptor) of a temperature at this value carries a greater evidence for the person’s continued existence than a measurement of −5°C. For a person to actively maintain themselves, it follows that they should actively seek out sensory data that provide self-evidence. It is this notion of self-evidencing that underwrites the first-principles account on offer here and is the same principle that has been used to reproduce a range of other phenomena in neuroscience (e.g., visual search behavior (Mirza et al. 2016), navigation and planning (Bruineberg et al. 2018; Kaplan and Friston 2018), curiosity (Friston et al. 2017b), reading (Friston et al. 2017d), action-observation (Friston et al. 2011), neglect syndromes (Parr and Friston 2017b), and hallucinations (Adams et al. 2013; Benrimoh et al. 2018; Parr et al. 2018)). This appeal to a common objective function (i.e., evidence for a generative model of the sensorium) across all these domains distinguishes the approach used here from alternative approaches to modeling behavior.

We can formalize this notion by defining some distribution over sensory observations, $P(\tilde{o})$ (where $\tilde{o}={\Big[{o}_1,{o}_2,\dots, {o}_T\Big]}^T$). The most probable observations are those that carry the greatest self-evidence and, a priori, constitute the preferred outcomes for the creature in question. The processes generating these sensations may be very complex, and so it is generally more efficient and tractable to evaluate a lower bound on the (log) evidence above and to seek to maximize the bound. This bound is the negative free energy (Dayan et al. 1995; Beal 2003):

$$-\underset{\mathrm{Free}\ \mathrm{energy}}{\underbrace{F\left(\pi \right)}}=\underset{\mathrm{Jensen}\hbox{'}\mathrm{s}\ \mathrm{inequality}}{\underbrace{E_Q\left[\ln \frac{P\left(\tilde{o},\tilde{s}|\pi \right)}{Q\left(\tilde{s}|\pi \right)}\right]\le \ln {E}_Q\left[\frac{P\left(\tilde{o},\tilde{s}|\pi \right)}{Q\left(\tilde{s}|\pi \right)}\right]}}=\underset{\log\ \mathrm{evidence}}{\underbrace{\ln P\left(\tilde{o}|\pi\right)}}$$

In machine learning and statistics, this is known as an evidence lower bound. Here, we have introduced the trajectories of hidden (i.e., latent) states (*s*) that cause sensory observations, and their dependence on policies (*π*) that denote sequences of actions. For the above inequality to hold, *Q* may be any arbitrary distribution. However, if we try to maximize the log evidence by minimizing the free energy (under the constraint that *Q* sums to 1), this distribution acquires an interesting interpretation. Rearranging the expression for the free energy gives the following:

$$F\left(\pi \right)={D}_{KL}\left[Q\left(\tilde{s}|\pi \right)\Big\Vert P\left(\tilde{s}|\tilde{o},\pi \right)\right]-\ln P\left(\tilde{o}|\pi\right).$$

The first term on the right-hand side means that the free energy is minimal when *Q* approximates the posterior distribution (Beal 2003). In short, self-evidencing appears to require perceptual inference, in the sense that a belief is formed that approximates the probability of the causes of sensations. Note that the term “belief” is used here in the technical sense of Bayesian belief updating—not to indicate a conscious, propositional belief. A convenient way to define *Q* is to use a mean field approximation (Feynman 1998):

$$Q\left(\tilde{s},\pi \right)=Q\left(\pi \right)\prod \limits_{\tau }Q\left({s}_{\tau }|\pi \right).$$

All that remains to define the free energy is to specify probability distribution over states and outcomes $P(\tilde{o},\tilde{s}|\pi)$, which is known as the generative model. This expresses the beliefs an animal has about the way in which its sensations or outcomes are generated by states of the world that finds itself in. The generative model can be factorized to give the following:

$$P\left(\tilde{o},\tilde{s}|\pi \right)=P\left({s}_1\right)\prod \limits_{\tau }P\left({o}_{\tau }|{s}_{\tau}\right)P\left({s}_{\tau +1}|{s}_{\tau },\pi \left(\tau \right)\right).$$

**Figure 1:**
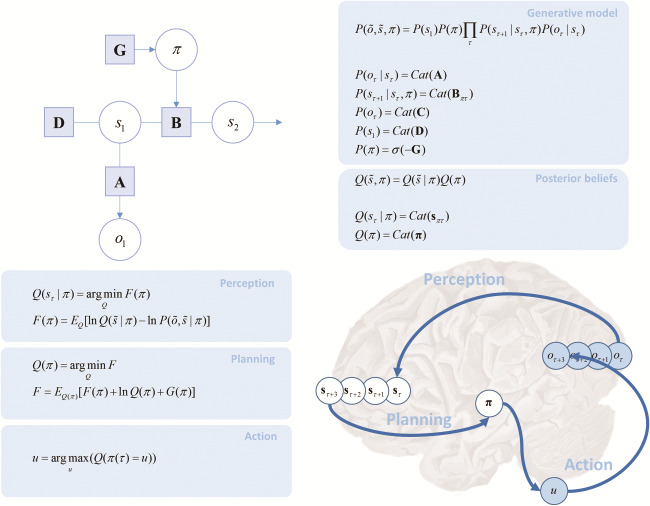
Markov decision process. The Bayesian network on the upper left shows the graphical representation of a Markov decision process. Technically, this is a partially observed Markov decision process, as the hidden states cannot be directly observed. Arrows between variables indicate conditional probabilities. The circles represent random variables that comprise observable outcomes (*o*), states (*s*), and policies, (*π*). Subscripts indicate time. The panel on the upper right shows the form of the (categorical) probability distributions that define this model. The prior distribution over policies is a softmax (normalized exponential) function of the expected free energy under each policy. The lower part of this figure summarizes active inference in terms of the reciprocal interactions between perception and action, following accounts of the prefrontal cortex as a temporal bridge in the perception–action cycle (Fuster 1990). On the left, we show the equations that describe this cycle. On the right, we illustrate this graphically, emphasizing the propagation of beliefs through time. Starting from the back of the brain, sensory areas send messages to higher regions encoding beliefs about the causes of those sensations. These beliefs are propagated forwards in time, allowing for a plan of action (Barceló and Cooper 2018) into the future (presumably evaluated in cortico-striatal loops). Once a policy has been inferred, this is used to select an action (*u*). Actions then cause changes in the physical world (e.g., movement of the eyes) that influence the sensory data obtained, allowing the cycle to start again. **G**, Expected Free energy; **D**, initial state prior; **B**, transition probabilities between hidden states; **A**, likelihood mapping from hidden states to outcomes. Please see Table 1 for a glossary of the variables used in this paper.

The form of these distributions is illustrated in Figure 1. This shows that there is a sequence of hidden states that evolve through time. At each time-step, these give rise to observable sensory data. The sequence of hidden states depends upon the policy pursued (Mirza et al. 2016). This formulation frames policy selection (planning) as a selection among different possible courses of action. Importantly, it also entails beliefs about the hidden states in the future and in the past. As we will see later, it is this temporal depth that underwrites working memory (Parr and Friston 2017d) as a key aspect of active inference. Although the equations in Figure 1 may look complicated, the generative model is relatively straightforward and generic in its form. The key parameters of this sort of model can be divided into **A**, **B**, and **C**. The **A** matrix describes the likelihood of any outcome given a hidden state, while a series of **B** matrices encode the transition probabilities among different hidden states (that depend upon the policy in play). Finally, the **C** matrix specifies prior beliefs about outcomes that underwrite self-evidencing.

**Table 1:** Glossary of variables

Variable	Definition
$\mathbf{F}$	Free energy
$\mathbf{G}$	Expected free energy
$\boldsymbol{\pi}$	Policy posterior
${\mathbf{s}}_{\pi \tau}$	State posterior belief (for a given policy and time)
${\mathbf{o}}_{\pi \tau}$	Outcome belief (for a given policy and time)
${o}_{\tau }$	Observed outcome
${\mathbf{A}}_{ij}=P({o}_{\tau }=i|{s}_{\tau }=j)$	Likelihood matrix (mapping states to outcomes)
${\mathbf{B}}_{ij}(u)=P({s}_{\tau +1}=i|{s}_{\tau }=j,\pi (\tau)=u)$	Transition matrix (mapping states to states)
${\mathbf{C}}_{\tau i}=P({o}_{\tau }=i)$	Outcome prior
${\mathbf{D}}_i=P({s}_1=i)$	Initial state prior
${\mathbf{H}}_i=\sum \limits_jP({o}_{\tau }=j|{s}_{\tau }=i)\ln P({o}_{\tau }=j|{s}_{\tau }=i)$	Entropy of the likelihood mapping

**Figure 2:**
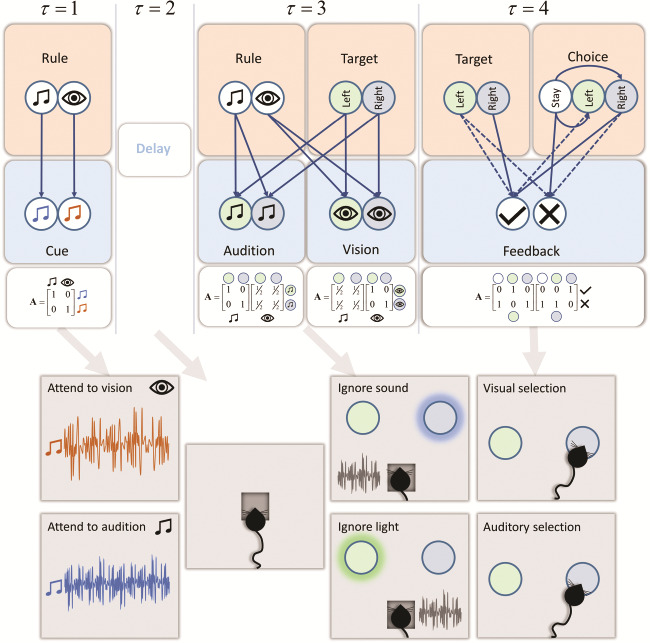
The generative model. The generative model we have used has a likelihood distribution that evolves over time. In this schematic, we illustrate the conditional dependencies between hidden states (pink panels) and sensory observations (blue panels) at each time point. During the delay period, sensations are conditionally independent of all states (i.e., there are no informative sensory data). The likelihood (**A**) matrices are shown below each sensory modality. All transition (**B**) matrices are identity matrices, except for the choice state. This depends upon the action selected by our synthetic mouse and allows it to transition from “no choice” (white circle) to the green or blue choices. Preferences (**C** matrix) are uniform for all outcome modalities, except the feedback modality. There is a preference, at the fourth time-step, for being correct over being incorrect. The initial prior beliefs (**D**) for all but the choice state are uniform. There is a prior belief that the first choice state will be the “no choice.” In practice, we model the time-dependency of the A matrix by conditioning it on an additional (time) hidden state, not shown here. The lower part of the schematic shows the course of a single trial. At the start, the mouse is presented with an auditory cue (blue or brown noise). This indicates either the “attend to vision” or the “attend to audition” rule. After a delay period, visual and auditory stimuli are presented. The mouse ignores the irrelevant stimulus and attends to the relevant one, ensuring it makes the correct choice, receiving positive feedback (milk). A failure to choose or the incorrect choice leads to negative feedback (no milk).

The best policies to select are those that lead to the lowest free energy in the future. To ensure that this is the case, we define a prior belief that the smaller the expected free energy, the greater the probability of pursuing that policy. The expected free energy is defined as follows:

$$\begin{align*}G\left(\pi \right)&={E}_{\tilde{Q}}\left[\ln Q\left(\tilde{s}|\pi \right)-\ln P\left(\tilde{o},\tilde{s}\right)\right]\\ &\approx-{E}_{Q\left(\tilde{o}|\pi \right)}\left[{D}_{KL}\left[Q\left(\tilde{s}|\tilde{o}\right)\Big\Vert Q\left(\tilde{s}|\pi \right)\right]\right]-{E}_{\tilde{Q}}\left[\ln P\left(\tilde{o}\right)\right]\\ {}\tilde{Q}\left(\tilde{o},\tilde{s}|\pi \right)&=Q\left(\tilde{s}|\pi \right)P\left(\tilde{o}|\tilde{s}\right)\end{align*}.$$

Note that we have augmented the approximate posterior used for the expectation so that it now includes an expectation over yet-to-be-observed sensory data. This acknowledges the complex interplay between action and sensation (Busse et al. 2017). The final term in the second line is the expectation of the values in the **C** matrix that specify prior preferences about outcomes—as defined in Figure 1. It is these prior beliefs that ensure the mouse will behave so that it ends up fulfilling its prior preferences. The first term mandates exploratory behavior, which is important in some contexts (Friston et al. 2015; Parr and Friston 2017c), but less so for the current task—as our task does not permit any foraging.

The representation of policies as deep temporal sequences of actions resonates with the notion that the prefrontal cortex acts as a temporal bridge in the perception–action cycle (Fuster 1990). Figure 1 illustrates this idea in relation to the formal perception–action cycle implied by active inference—and summarizes the discussion above. In the next section, we define the generative model used for our task and appeal to the process theory (Friston et al*.* 2017a, 2017c) associated with active inference to describe the requisite neuronal message passing (Winn and Bishop 2005; Dauwels 2007). In brief, this (variational) message passing involves passing sufficient statistics between neurons or neuronal populations. The sufficient statistics encode posterior beliefs about things that need to be inferred, namely, hidden states of the world and the policy being pursued. Here, the sufficient statistics are just the expected probability of being in a particular state—or pursuing a particular policy. The requisite message passing can be formulated as a gradient descent on variational free energy, which provides a plausible account of the neuronal dynamics.

The neuronal processing scheme adopted in this work may appear a little complicated and ad hoc. However, unlike alternative schemes (e.g., policy optimization) this is a “first principle” scheme that optimizes a single quantity (a variational bound on Bayesian model evidence). It is important to appreciate that this (message passing) scheme has been used in a series of studies, reproducing a wide range of phenomena, from working memory and attention during saccadic eye movements through to epistemic foraging in T-mazes—from evidence accumulation in neuroeconomics games to simulating curiosity and rule learning (Moutoussis et al. 2014; Friston et al*.* 2015, 2017b; Mirza et al. 2016). The only thing that differs among these (and the current) applications is the implicit model of how states of the world generate outcomes. Furthermore, the implicit message passing is neuronally plausible, relying upon a series of linear mixtures and nonlinear transformations (Friston et al. 2017d; Parr et al. 2019). This belief updating for generative models of discrete states (i.e., hidden Markov models and partially observable Markov decision processes) can be regarded as equivalent to predictive coding for continuous states (i.e., state space models). The formal equivalence is discussed at length in the setting of the graphical brain (Friston et al. 2017c).

**Figure 3:**
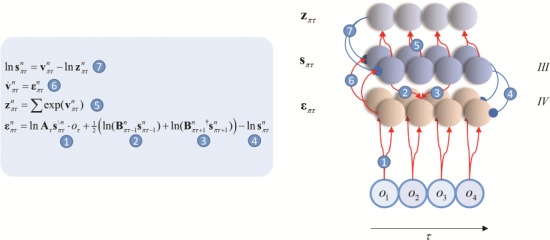
Intrinsic connectivity and neuronal message passing. The equations on the left show the form of the variational message passing mandated by active inference. On the right, we represent these in the form of a neuronal network. Blue connections are inhibitory, and red are excitatory. The input layer is labeled layer IV for consistency with patterns of laminar connectivity in the cortex (Zeki and Shipp 1988; Felleman and Van Essen 1991; Shipp 2007). Layer III houses the cells representing the sufficient statistics of posterior beliefs. It is these that are connected through di-synaptic recurrent excitatory connections and lateral inhibitory connections. The notation ${\mathbf{s}}_{\pi \tau}^n$ means the expected hidden state factor *n* at time *τ*, conditioned on a policy. The quantity **z** is a normalizing constant (or partition function), while **v** and **ε** are auxiliary variables that play the roles of membrane depolarization and prediction error, respectively. The network illustrated here is supposed to show how populations of neurons interact with one another to perform variational inference. This implies a representation of beliefs in terms of population averages (or other population codes). While we have shown excitatory and inhibitory connections arising from the same populations, this is not intended to imply a violation of Dale’s law, but instead implies intermediate inhibitory interneurons (that are omitted for clarity).

## The Generative Model

The simulations used in this paper are based upon the implementation of active inference—under Markov decision processes—described above. This implementation has been used to simulate a large range of electrophysiological and psychophysical phenomena in visual neuroscience (e.g., saccadic eye movements), through to higher cognitive functions (e.g., abstract rule learning). Each application uses exactly the same computational architecture and message passing scheme. The unique aspect of each application rests upon the particular generative model appropriate for the task or paradigm at hand. In this section, we consider a minimal generative model that is apt for the task we have chosen to characterize context-sensitive delay-period activity.

The task is structured as illustrated in the lower part of Figure 2 (Wimmer et al. 2015). First, an auditory cue is presented. This is followed by a delay period and then simultaneous auditory and visual target stimuli. Depending upon the identity of the predelay cue, the mouse should attend to its visual or auditory modality after the delay. The cued modality tells the mouse whether it should choose the left or right option. Following its choice, the mouse is given feedback (in the form of milk or no milk). In Figure 2, we show the form of the generative model required to perform the task described above. There are 3 types of hidden state. These are the rule, the target, and the choice. The mapping from each of these hidden states to sensory outcomes changes with each time-step. Initially, there is an identity mapping between rule states and the cue. During a delay period, there are no sensory data generated (i.e., each modality constitutes a “null” or uninformative outcome). At the third time-step, visual and auditory stimuli are generated from the target state, but in a way that depends upon the rule. If the rule is “attend vision,” there is an identity mapping from the target to the visual modality and an uninformative mapping to the auditory modality. These mappings are reversed if the rule is “attend audition.” These mappings of the generative model express the beliefs that the attended modality contains useful information, while the unattended modality is imprecise and is uninformative about the correct target. During the final (fourth) time-step, feedback is given. This depends upon the choice made and on whether it matches the correct target. A failure to make a choice leads to an incorrect outcome. A priori, our simulated (murine) subject expects to be correct (and receive milk). This means that it infers it will follow the course of action most consistent with this prior belief.

While the generative model we have employed here is clearly specific to the task in question, it includes several features commonly found in cognitive tasks (Funahashi et al. 1989; Griffin and Nobre 2003; Lepsien and Nobre 2007; Astle et al. 2009; Lepsien et al. 2011; Pertzov et al. 2013). Most obviously, the propagation of beliefs through time—such that beliefs about states early in a multi-step sequence influence beliefs about states later on. The sort of message passing required to perform this kind of task, regardless of the specific outcome modalities, must include a similar transition structure, where beliefs about states in future may be confidently predicted based upon those in the past or present. This suggests that, while the form of the A matrices and the sensory data they represent may vary from task-to-task, the need for precise B matrices—and their associated recurrent connectivity (Fig. 3)—may be a more generic feature of sequential processing in working memory tasks.

## Anatomy

### Intrinsic Connectivity

To maintain a representation of something beyond the duration of a stimulus presentation, 2 things are crucial. The first is persistence of neuronal activity—this is often referred to as “delay-period” activity (Funahashi 2015). The second is a clear separation between alternative representations (i.e., the “tuning” of memory cells; Murray et al. 2014). The latter is vital for the specificity of the former. These 2 properties have been investigated extensively through electrophysiology and anatomical tract tracing, and the neurobiological machinery that support each has been well characterized (Coull 1998; Goldman-Rakic 2011; Arnsten 2013). Delay-period activity is supported by recurrent glutamatergic connections within layer III of the lateral prefrontal cortex (Kritzer and Goldman-Rakic 1995). This ensures populations of pyramidal cells may remain persistently active through local (at most di-synaptic) connectivity (Constantinidis et al. 2001). The tuning of prefrontal pyramidal cells depends upon inhibitory interneurons (Constantinidis and Goldman-Rakic 2002) and is destroyed by GABA antagonists (Rao et al. 2000).

Interestingly, these 2 features turn out to be emergent properties of a system that engages in active inference. Figure 3 shows the form of the neuronal message passing required for inference about states under the generative model in Figure 1 (Friston et al. 2017d). This exhibits recurrent excitatory (di-synaptic) connections and inhibitory interneurons—and affords a computational interpretation of their function. Excitatory connections (labeled 2, 3, and 6) reflect the fact that beliefs at one time should inform beliefs about events in both the future and the past. The strength (or gain) of these connections determines the degree to which beliefs about the past constrain the present, and vice versa (and similarly for the present and the future). Technically, this manifests in a generative model as a precision (inverse variance) of beliefs about transitions. To maintain a belief over time, as required in this task, this precision must be sufficiently high. In the section on neuropsychology later, we will see the effect of disrupting this precision on maintenance of internal representations over time. A set of inhibitory connections (labeled 7) act to ensure that 2 opposing beliefs cannot be held simultaneously. In other words, if one possibility becomes more probable, it must be the case that alternatives become less so. It is easy to see how the loss of this constraint (through loss of GABAergic signaling) results in the loss of precise tuning curves (Rao et al. 2000).

### Extrinsic Connectivity

Above, we described the intrinsic connectivity within a prefrontal region and the computations it could support. We now turn to the connectivity between regions of prefrontal cortex and consider how the computations described above may align with functional anatomy. This should not be taken too seriously from an anatomical perspective but provides a useful vehicle to unpack the sorts of computational architectures implied by the generative model used here, particularly with reference to the influence of different hidden state factors over one another. Furthermore, it will be useful in formalizing the influences of various synthetic lesions in an anatomically informed manner in the neuropsychology section below. Prefrontal regions, in primates, have a topography defined in part by the patterns of input from other areas. Loosely speaking, in the lateral part, ventral areas tend to receive input from the “what” pathways (i.e., information about identity from ventral visual and auditory areas), while the dorsal regions receive input from “where” pathways (carrying spatial information; Wilson et al. 1993; Romanski et al. 1999). The visual and auditory stimuli in our generative model, and the choices, may be interpreted either as representing 2 alternative spatial locations or as 2 alternative objects. Although the intrinsic connectivity remains identical, the former speaks to a dorsal representation and the latter to engagement of ventral prefrontal circuitry.

Medial parts of the prefrontal cortex, including the orbitofrontal and anterior cingulate regions (Barbas and García-Cabezas 2016), are the targets of structures associated with interoceptive sensation (Price et al. 1996; Groenewegen and Uylings 2000; Ghashghaei and Barbas 2002). Together, these observations imply a dorsal–ventral axis representing location to identity (sometimes characterized as “how” and “what”; O’Reilly 2010) and a lateral–medial axis representing exteroceptive to interoceptive terminations (sometimes called “cold” and “hot”; O’Reilly 2010). Some authors additionally propose a caudal to rostral axis corresponding to levels of hierarchical abstraction (Badre and D'Esposito 2009). Given that higher levels in a hierarchy tend to represent states that evolve over longer time periods (Hasson et al. 2008), this is highly consistent with recent findings that training in working memory tasks induces plastic changes in more anterior (i.e., abstract) parts of the prefrontal cortex (Riley et al. 2018).

Clearly this is a caricature of prefrontal anatomy (e.g., orbitofrontal regions are also in receipt of exteroceptive information; Barrett et al. 2013), but it is a useful one as it affords the opportunity to form empirical hypotheses about the electrophysiological responses in different regions of prefrontal cortex and makes predictions about the sorts of deficits expected following lesions that are answerable to neuropsychological data. For more detailed and nuanced accounts of prefrontal organization, please see Price et al. (1996), Barbas and Zikopoulos (2007), Barbas (2015), and Nee and D’Esposito (2016).

The task we have described depends upon all of the variables outlined above, as the rule is cued through 1 of 2 distinct sounds, the stimuli to attend to are visual and auditory, and the feedback (milk) has interoceptive consequences. The former areas require inputs from visual and auditory areas, implicating the lateral part of the cortex. The latter needs input from gustatory regions, such as the insula cortex, or regions representing emotionally salient stimuli, such as the amygdala, suggesting medial prefrontal areas (Mufson et al. 1981; Devinsky et al. 1995; Gu et al. 2013). This is consistent with the suggestion that medial prefrontal areas, including the orbitofrontal cortex, might be involved in “value”-based inferences (Wallis 2011) or in representing action–outcome contingencies (O’Callaghan et al. 2018).

**Figure 4:**
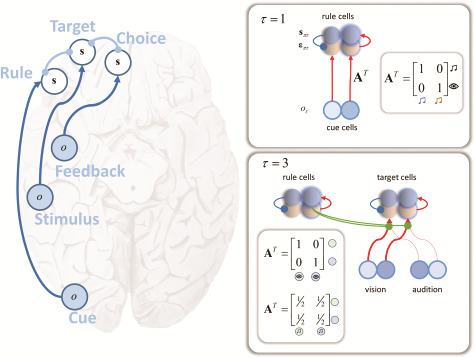
Extrinsic connectivity. The schematic on the left illustrates the types of input that reach different parts of the prefrontal cortex. Exteroceptive modalities tend to target lateral parts, while structures associated with interoceptive inference (Seth 2013; Barrett and Simmons 2015) project to more medial regions. These inputs may be informative about more than one hidden state. This is expressed through modulatory connections between regions that act to contextualize the incoming sensory information (term 1 in the equations in Fig. 3). On the right, we provide an example of this. At the third time-step, immediately after the delay period, the input to target cells is given a context by the beliefs about the rule (derived from the sensory input 2 time-steps previously). This controls the gain of connections from each sensory modality to the target cells. Technically, this interaction occurs due to the fact that the rule and the target occupy each other’s Markov blankets (in virtue of their jointly causing outcomes). This implies a reciprocal interaction, such that regions representing one should connect to those representing the other, and vice versa. Anatomically speaking, this means that prefrontal regions that interact with one another should show bidirectional connectivity. These connections may be between hierarchical levels (Kiebel et al. 2008), or may be lateral modulatory influences.

The interaction between beliefs about different hidden state factors depends upon their jointly generated sensory outcomes, i.e., sitting within each other’s Markov blanket as the parents of the same children (Pearl 2014). The cue stimulus, before the delay, depends only upon the rule state. In contrast, the visual and auditory cues depend upon both the rule and the target (Fig. 4). This means that, during perceptual inference, beliefs about the rule should contextualize the mapping from visual and auditory modalities to the cells representing the target. Similarly, the feedback depends upon both the target and the choice, implying the 2 should modulate one another. As illustrated in Figure 4, these conditional dependencies suggest regions representing rules and targets should modulate one another, as should those representing targets and choices. Note the qualitative correspondence between the connectivity between these 3 sets of beliefs (about each hidden state factor) and the anatomical patterning of the 3 modes of activity identified with different stages of a working memory task (Markowitz et al. 2015).

**Figure 5:**
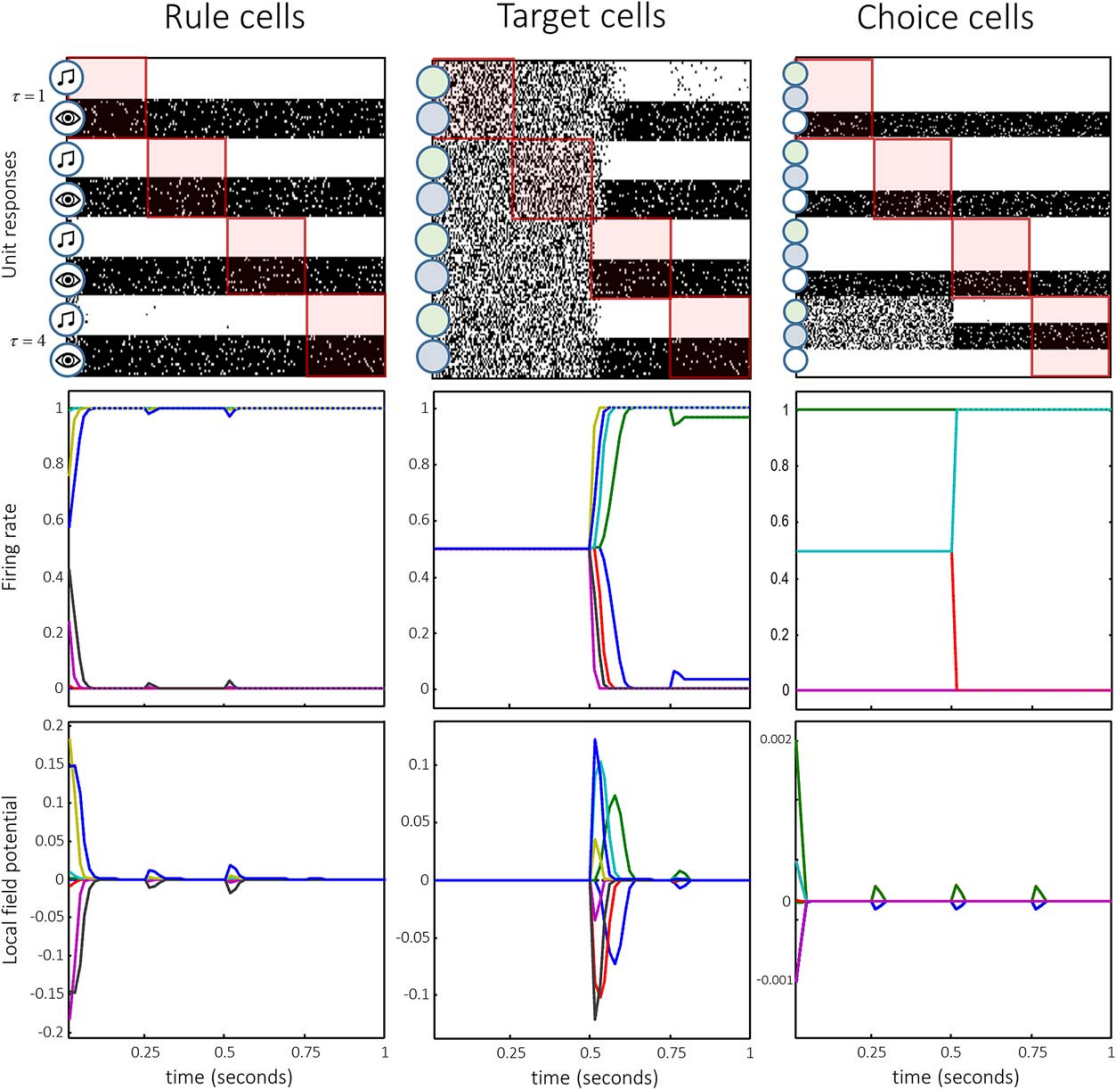
Simulated electrophysiology. These plots show the electrophysiological responses simulated for each of the 3 unit representations during the trial shown in Figure 1. The upper row shows the responses we would expect to record from single cells in these populations. These are presented in a “raster plot” format, with higher firing rates represented as denser regions. Each column within these plots represents a population of neurons. Neurons shown at the top of the column encode beliefs about the first time-step in a trial. Those at the bottom encode beliefs about the final time. The red boxes indicate the neurons that encode beliefs about the present. Those above these boxes encode beliefs about the past, and those below encode beliefs about the future. The second row of plots shows the same information in a slightly different format. Each line represents a different neuron (i.e., a row from the raster plot) and shows the trajectory of firing rates over time. The lower row of plots shows the simulated LFPs. All 3 rows are temporally aligned.

Figure 4 illustrates the form of the modulatory influence rule cells exhibit over the connections from visual and auditory modalities. This mediates a form of context-dependent gain control (Parr and Friston 2017c) analogous to that used to account for endogenous attention (Feldman and Friston 2010). In brief, attention to 1 of the 2 sensory modalities is the process of ascribing a greater precision to the likelihood distribution mapping hidden states to that type of outcome. When the rule “attend to vision” is inferred, this leads to the deployment of an identity likelihood matrix (that is infinitely precise) generating visual data, and a uniform (zero precision) distribution generating auditory signals. The latter renders beliefs about targets conditionally independent from auditory inputs. An inference that the rule is “attend to audition” would reverse these matrices. The modulation of these inputs by prefrontal regions is consistent with empirical work that highlights the importance of such regions in the coordination of attention (Rossi et al. 2008).

## Electrophysiology

In this section, we solve the equations in Figure 3 (and the appendix), through simulation, for the task outlined above. The form of these equations allows us to associate variables with idealized electrophysiological measurements (Friston et al. 2017a). The auxiliary variable **v** plays the role of a postsynaptic potential, computed from the inputs from other neurons. This is transformed to **s**, and it is this signal that is propagated to other neuronal populations, analogous to a firing rate. This allows us to associate the rate of change of **v** with the local field potentials (LFPs) caused by depolarizations, and **s** with the signal measured through single unit recordings. Figure 5 shows these responses for each population of neurons (those representing the rule, target, and final choice).

The key aspect of these synthetic neuronal responses rests upon the deep temporal models used to update beliefs and accumulate evidence for the time and context-dependent states of the world. The very fact that our simulated mice can remember and plan rests upon the representation of the past and future in terms of (the sufficient statistics of) posterior beliefs—as encoded by neuronal firing. This suggests there is a “place coding for time” in the prefrontal cortex, or indeed any part of the brain that participates in the inversion of deep temporal models. A subtle but instructive aspect of this implicit short-term memory—for the past and future—rests upon the way sensory outcomes are generated over a given epoch of time (e.g., the successive phases of a trial). By construction, the inference scheme assigns representations to various time points from the beginning to the end of an epoch. This means that at the beginning of a trial most neurons (or populations) are encoding posterior beliefs about the future, while at the end of the trial they encode posterior beliefs about the past—which have been updated on the basis of sensory evidence. This equips the mice with the capacity to both predict and postdict as anticipation turns into short-term memory, as each neuron’s designated time approaches and passes. Figure 5 illustrates this in terms of simulated neuronal responses that are plotted with the time through the trial along the *x*-axis and the time represented by the neurons along the *y*-axis.

The implication here is that delay-period activity is a necessary and emergent property of any belief updating based on models that generate the sensory consequence of actions that, by definition, can only occur in the future. Furthermore, the context sensitivity of these consequences necessarily implies a modulation of (synaptic) coupling that determines the onset an offset of delay-period activity. On this view, delay-period activity is neither purely anticipatory, nor purely mnemonic. It represents the accumulation of evidence for various states of the world that provide a context in which different sorts of latent or hidden states are updated distinct neuronal populations.

In our simulations, the time course of the responses in each neuronal population is different, consistent with empirical data (Fuster 1973; Fuster et al. 1982; Koechlin et al. 2003; Hunt et al. 2015). This suggests it should be possible to use electrophysiological data to discriminate between the neurons playing each role. Note that the rule cells rapidly increase or decrease their firing rates at the time of the cue presentation, consistent with measured cells in the rodent prefrontal cortex (Schmitt et al. 2017), and with human neuroimaging data (Woolgar et al. 2011). This is accompanied by a large LFP. They maintain persistent activity throughout the delay period and remainder of the trial (Funahashi et al. 1989). Target cells, representing the correct option, become active when the visual and auditory stimuli are presented. They should respond to a particular auditory stimulus for 1 rule (and should be unresponsive to visual stimuli), with the same cells responding to a particular visual stimulus when the other rule is in play.

The simulated electrophysiology shown in Figure 5 serves 3 key purposes. First, it lends a construct validity to the inferential perspective (and generative model) we have used, in the sense that these results would not be unexpected in real electrophysiological measurements. Second, it offers an intuition as to the belief-updating process used by the simulated mice to solve the task. This will be important for the neuropsychology section that follows, where changes to the generative model (i.e., synthetic lesions) have consequences for how this belief-updating unfolds. Finally, this means we can derive quantitative predictions about measured electrophysiology from animal behavior and use these predictions to investigate the representation of beliefs in prefrontal cortical (and subcortical) circuits.

**Figure 6:**
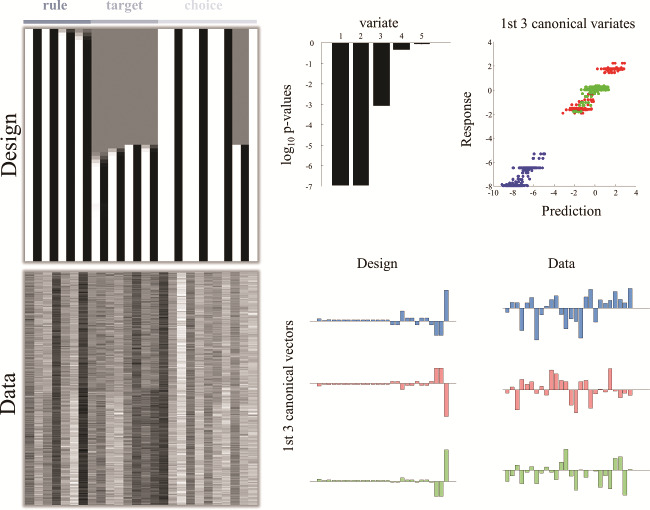
An example analysis. This figure illustrates how this approach could be used to assess the predictive validity of the model in relation to empirical data. This example uses a standard multivariate approach known as Canonical Variates Analysis (CVA) to test whether some linear mixture of explanatory variables in a design matrix predicts some pattern (mixture) of multivariate data. The basic approach is shown above for a simulated analysis. This is intended to illustrate how one might employ this model in an empirical setting (i.e., no interpretations should be drawn from the synthetic results shown here). The design matrix shows the simulated (average) firing rates for the neurons representing rules, targets, and choices for an example trial (as in Fig. 5). This could be generated using parameters estimated from behavioral data (crucially, not from the electrophysiological data). Each column represents a neuronal population, and each row represents a time-step through the trial. The “data” matrix here is constructed by multiplying the design matrix with a randomly generated matrix, so that each column of the data matrix is distinct linear mixture of the columns of the design matrix (i.e., emulating a distributed representation of beliefs through time) and adding some noise (with the signal-to-noise ratio of 8). In a real analysis, the data matrix would be constructed from measured neural time-series (following averaging and smoothing), where each column is a neuron, and each row a time-bin. In our example, CVA finds evidence in favor of 3 canonical variates that each represent a linear mixture of the data and a linear mixture of the simulated data. As shown in the upper right plot, one can now assess the degree to which each pattern of the belief trajectories predicts its associated pattern of observed responses (i.e., the neural correlates of belief updating). The weights shown in the canonical vectors constitute a profile of responses that enable inferences about functional segregation (e.g., a pattern that implicates only those neurons from a particular sub-region of the prefrontal cortex).

An advantage of being able to simulate behavior—and the electrophysiological correlates of the inferential processes that underwrite this behavior—is the opportunity to assess the predictive validity of these models, against empirical behavior. By fitting the model outlined above to behavioral (choice) data (see, e.g., Mirza et al. 2018), we can estimate the parameters of the generative model (i.e., prior beliefs) for individual animals. These can then be used to generate simulated electrophysiological data of the sort shown in Figure 5, specific to a given mouse, which are then answerable to real data recorded from the prefrontal cortex of the same mouse. This means we can ask whether it is possible to predict neuronal responses based upon observed behavior. Figure 6 provides a simple example of how this sort of analysis could be performed.

**Figure 7:**
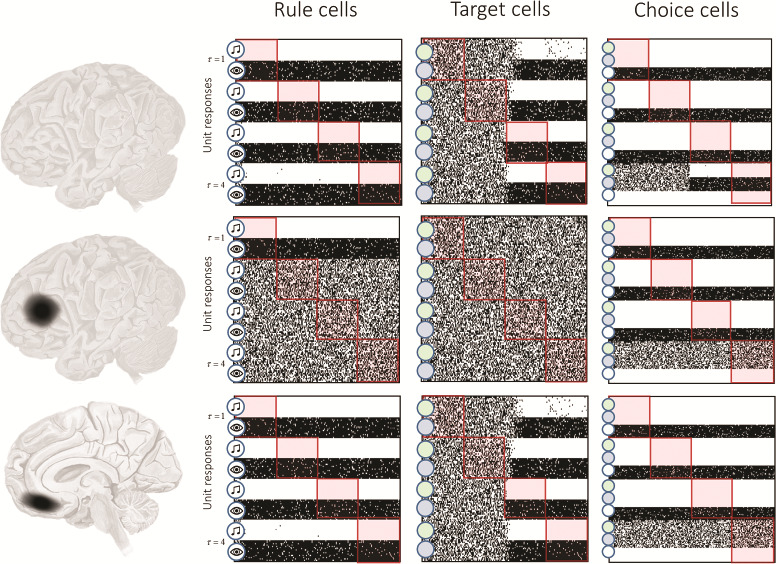
Synthetic prefrontal lesions. In the upper row of this figure, we reproduce the simulated raster plots from Figure 5. In the middle and lower row, we show raster plots of the same cells during the same trials (brown noise, followed by an auditory cue to the blue target), but with lesions to the lateral or medial prefrontal cortex. Lesions to the lateral cortex (middle row) were simulated by using uniform distributions for the transition probabilities associated with the rule hidden state. This effectively disconnects the $\mathbf{B}$-connections (Fig. 3) for this cell population. Medial lesions (lower row) were simulated by setting the $\mathbf{A}$-connections from the feedback outcomes to uniform distributions. This disconnects interoceptive regions from the medial prefrontal cortex.

The simulations in this section assume that the generative model is a good description of the way in which stimuli are presented to the mouse throughout the task, i.e., they comply with the good regulator theorem (Conant and Ashby 1970). This means the simulated mice do not make any errors. However, real mice are much more likely to get things wrong, and it is important to account for this. The electrophysiological responses shown here provide one way of looking at this. For example, it is clear that a failure to maintain delay-period activity would make it impossible to infer the correct choice at the end. This would lead to a random choice between the 2 alternatives, with errors on half of the trials (if this were a complete failure to maintain this delay period). This raises the following question: what would need to change in the set-up of the generative model for Bayes optimal inference to fail to propagate beliefs about the rule forwards through time?

The answer to this question comes from the IntrinsicConnectivity section above, which discusses the recurrent glutamatergic connections that maintain delay-period activity. Figure 3 equates these connections with the B matrix that constrains the future based upon the present. This implies that these connections, and therefore the delay-period activity, will be attenuated when the past is believed to be less predictive of the present (i.e., in the presence of imprecise dynamics). This highlights the importance of precise beliefs about temporal dynamics in order to contextualize beliefs about the present (c.f. “distrusting the present”; Hohwy et al. 2016). We will see an extreme example of this failure in the Neuropsychology section below. More concisely, performance errors result from a generative model that does not reflect how the data presented to the mouse were actually generated. This is not to say that such models are suboptimal. Clearly the future cannot always be predicted perfectly, based upon the present; so, it may be that imprecise prior beliefs about environmental dynamics are perfectly consistent with the good regulator theorem, when considering the data mice contend with, while not performing this task.

Having established a degree of construct validity, in relation to empirical delay-period activity and its context-sensitive aspects, we now consider the implications of this form of neuronal belief updating and evidence accumulation for neuropsychology.

## Prefrontal Neuropsychology

Lesions to the prefrontal cortex in humans have been associated with various, anatomically sensitive, cognitive impairments (Szczepanski and Knight 2014). Lateral lesions tend to cause impairments at delayed response tasks, like that we have described here (Passingham 1985). Medial (and ventromedial) lesions have a very different profile (Harlow 1999). Case studies of patients with these lesions (Papez 1937; Eslinger and Damasio 1985; Damasio et al. 1994) reveal a phenotype that is distinct from that associated with lateral lesions. Despite retaining a high level of intelligence—and being unimpaired on classic tests of frontal lobe function—patients generally lack spontaneous motivation. The same behavioral phenotype can be found in patients with lesions in subcortical structures projecting to this part of cortex (Adam et al. 2013). In this section, we bring together the ideas we have presented above and simulate lesions to the lateral and medial parts of our in silico prefrontal cortex. We do so by disrupting 2 components of the generative model. Lesions to the lateral prefrontal cortex are simulated through disruption of the (intrinsic) recurrent excitatory connections that we have associated with rule transition probabilities. To simulate medial lesions, we disconnect the “feedback” input, simultaneously mirroring the white matter disconnections that can induce a medial prefrontal syndrome (Van Horn et al. 2012) and the “insensitivity to future consequences” (Bechara et al. 1994) associated with these patients.

Figure 7 shows the consequences of these lesions for the trajectories of beliefs throughout a trial. Following a lateral lesion, the recurrent connectivity that propagates beliefs about a given time to future time steps has been disrupted. This means that, as soon as the delay period begins (i.e., the cue is removed), the belief about the current rule becomes uniform. This results in a failure to modulate the gain of visual and auditory inputs following the delay. When opposing stimuli are presented following the delay, this leads to a failure to infer both the target and the correct choice. Medial lesions spare inferences about rules, their propagation to cells representing future times, and correct contextualization of visual and auditory stimuli, resulting in a correct inference about the target. However, inferences about the choice remain very uncertain. This is consistent with the preservation of intelligence and the ability to perform this kind of task, but the apathy and unusual choices made by patients with these lesions (Bechara et al. 1998; Bechara 2004). An inability to make inferences based on feedback might also account for the perseverative errors observed in patients with medial prefrontal lesions (Freedman et al. 1998). Perseverative deficits are also observed in patients with lateral lesions (Nyhus and Barceló 2009), but our simulations suggest that this could be due to a failure to propagate beliefs through time to induce learning, as opposed to a failure to use feedback to update beliefs.

We note that there are other plausible lesions we could have made to induce these kinds of deficits. For example, in place of the intrinsic disconnection we induced among cells representing the rule, we could have performed an extrinsic disconnection, rendering the rule and cue conditionally independent of one another. In place of the medial disconnection, we could have disrupted the prior preferences. This speaks to the possibility that biological lesions may compromise multiple computational mechanisms, individually or together, to induce these deficits. The fact that it is possible to specify a range of plausible lesions means that we have a space of hypotheses, expressed in terms of the priors accompanying a generative model. This offers a computational tool to try to disambiguate between these hypotheses through model comparison, using patient choice behavior (Schwartenbeck and Friston 2016; Mirza et al. 2018). This provides an opportunity to use the same model to assess behavior in mice, where detailed electrophysiology and optogenetic manipulations are available, and to answer questions about the computational deficits that underwrite human neuropsychological syndromes.

## Discussion

The key insight that arises from this approach is that the sort of computational architecture found in the prefrontal cortex closely resembles the anatomy required for inference under deep temporal models. Specifically, this circuitry is prescribed by the message passing that solves a minimal generative model of a task used to investigate prefrontal cortex. This formulation has a practical utility, because choice behavior, and its electrophysiological correlates, can be predicted from the same model; by appealing to the Bayesian belief updating used to infer the appropriate course of action. This offers a formalism to assess the functional circuitry of prefrontal inference in mouse models, where both behavior and electrophysiology can be recorded simultaneously. Put simply, the choices made by a mouse in a given trial constrain the inferences that must have taken place to give rise to those choices. Comparing these inferences with single unit recordings from different parts of prefrontal networks affords an opportunity to associate these cells with their computational analogues in the network shown in Figure 3.

Given that we have focused on a task for which animals are typically over-trained, we have assumed that the structure of the generative model and its contingencies have been learned in advance and therefore do not simulate this learning process. However, it is possible to appeal to the same variational principles used to simulate inference to derive the Bayes optimal updates to beliefs about the parameters of a generative model (via accumulation of Dirichlet parameters). The form of these updates (Friston et al. 2016) turns out to resemble activity-dependent plasticity. For example, in the context of the likelihood distribution, the probability of an outcome given a state is increased whenever the 2 co-occur, just as the co-occurrence of pre and postsynaptic depolarization leads to synaptic potentiation (Bliss and Lomo 1973).

This is an important direction to pursue for 2 reasons. First, this will be crucial in understanding the processes that underwrite plastic changes in prefrontal cortex during working memory training (Riley et al. 2018). Second, it will be important in accounting for certain types of error or bias in these kinds of task. We could distinguish between 3 sorts of error. One which is due to false inference, where the wrong distributions are learned. This could be due to poor quality training data, or to inappropriate prior beliefs about the parameters. The second is that the mice learn (correctly) based upon a small number of trials that they are more likely to select a given choice. This prior belief would bias future actions (even when the rewarded choice is fully stochastic) and reinforce this behavior, i.e., habit formation (FitzGerald et al. 2014). Finally, the mice might behave for reasons other than achieving the milk. As we highlighted in the Active Inference section above, policies that minimize expected free energy are not always exploitative. There is also value in exploration or information gain (Itti and Baldi 2006). Given uncertainty about the contingencies of the task, optimal behavior could include trying to find out what would happen given a choice (even if the mouse is confident that the other choice would lead to milk).

## Conclusion

In the above, we demonstrated that the variational solution to a rule-guided selected attention task requires a computational architecture that bears a close resemblance to prefrontal cortical functional connectivity. This facilitates an interpretation of these connections in terms of the inferential functions they support. In short, recurrent intracortical excitation might reflect beliefs about transitions, while lateral inhibition acts to normalize beliefs about alternatives. The lateral-to-medial axis of the primate prefrontal cortex appears to reflect an exteroceptive-to-interoceptive axis and accounts for different phenotypes corresponding to anatomically distinct prefrontal regions.

Perhaps unforgivably, we have neglected 2 important features of prefrontal function. We hope, in future work, to address the inferential role of the mediodorsal thalamus (Rikhye et al. 2018), projections from which define prefrontal cortex. Disruption of activity in this nucleus prevents the performance of the above task, while stimulation enhances it (Schmitt et al. 2017). We also aim to understand the perseverative errors that prefrontal lesions can induce. These reflect a failure of reversal learning, and the plastic changes underwriting this should feature in any comprehensive description of the prefrontal cortex. Despite these omissions, our simulations revealed a high degree of face validity, reproducing delay-period responses that are abolished by lesions to lateral intrinsic connectivity. Furthermore, they make clear predictions about the sorts of signals that can be measured in the prefrontal cortex and the consequences to these signals of focal anatomical lesions.

## Software note

Although the generative model changes from application to application, the belief updates described in this article are generic and can be implemented using standard routines (here spm_MDP_VB_X.m). These routines are available as Matlab code in the SPM academic software: http://www.fil.ion.ucl.ac.uk/spm/. Simulations of the sort reported above can be reproduced (and customized) via a graphical user interface by typing in >>DEM.

## A. Appendix

In this appendix, we give an outline of the derivations for the inferential belief update equations and for the equations used for policy selection. For more technical accounts please see (Friston et al. 2017a, 2017c).

### A.1. Inference

The free energy for a given policy is given as

$$F\left(\pi \right)={E}_Q\left[\ln Q\left(\tilde{s}|\pi \right)-\ln P\left(\tilde{o},\tilde{s}|\pi \right)\right].$$

If we are interested in finding $Q({s}_{\tau }|\pi)$, we can omit all terms that are constant with respect to this

$$\begin{align*} F\left(\pi \right)&={E}_{Q\left({s}_{\tau }|\pi \right)}\left[\ln Q\left({s}_{\tau }|\pi \right)\right]\\&\quad-{E}_{Q\left({s}_{\tau -1}|\pi \right)Q\left({s}_{\tau }|\pi \right)}\left[\ln P\left({s}_{\tau }|{s}_{\tau -1},\pi \right)\right]\\ {}\kern2em &\quad-{E}_{Q\left({s}_{\tau }|\pi \right)Q\left({s}_{\tau +1}|\pi \right)}\left[\ln P\left({s}_{\tau +1}|{s}_{\tau },\pi \right)\right]\\&\quad-{E}_{Q\left({s}_{\tau }|\pi \right)}\left[\ln P\left({o}_{\tau }|{s}_{\tau}\right)\right]. \end{align*}$$

In terms of sufficient statistics, this is

$$\begin{align*} {\mathbf{F}}_{\pi }&={\mathbf{s}}_{\pi \tau}\cdot \left(\ln {\mathbf{s}}_{\pi \tau}-\ln {\mathbf{B}}_{\pi \tau -1}{\mathbf{s}}_{\pi \tau -1}\right.\\ &\left.\qquad\qquad-\ln {\mathbf{B}}_{\pi \tau}\cdot {\mathbf{s}}_{\pi \tau +1}-\ln \mathbf{A}\cdot {o}_{\tau}\right). \end{align*}$$

We can then set up a gradient descent on this using an auxiliary variable, ${\mathbf{v}}_{\pi \tau}$:

$$\begin{align*} {\mathbf{s}}_{\pi \tau}&=\sigma \left({\mathbf{v}}_{\pi \tau}\right)\\ {}{\dot{\mathbf{v}}}_{\pi \tau}&=-{\nabla}_{\mathbf{s}}{\mathbf{F}}_{\pi }={\boldsymbol{\varepsilon}}_{\pi \tau}\\ {}{\boldsymbol{\varepsilon}}_{\pi \tau}&=\ln {\mathbf{B}}_{\pi \tau -1}{\mathbf{s}}_{\pi \tau -1}+\ln {\mathbf{B}}_{\pi \tau}\cdot {\mathbf{s}}_{\pi \tau +1}\\&\quad+\ln \mathbf{A}\cdot {o}_{\tau }-\ln {\mathbf{s}}_{\pi \tau}. \end{align*}$$

Here, $\sigma$ is a softmax (normalized exponential) function. Following this same line of reasoning, but for multiple factors of hidden state, and multiple outcome modalities, we reach the same equation as above, but replace $\ln \mathbf{A}\cdot {o}_{\tau }$ with $\sum \limits_m\ln {\mathbf{As}}_{\pi \tau}^{\backslash n}\cdot {o}_{\tau}^m$ to make inferences about ${s}_{\tau}^n$.

The mean field approximation here often leads to overconfident posterior beliefs. In practice, we compensate for this by using the following expression for the gradient (Parr et al. 2019):

$$\begin{align*} {\boldsymbol{\varepsilon}}_{\pi \tau}&=\frac{1}{2}\ln \left({\mathbf{B}}_{\pi \tau -1}{\mathbf{s}}_{\pi \tau -1}\right)+\frac{1}{2}\ln \left({\mathbf{B}}_{\pi \tau}^{\dagger }{\mathbf{s}}_{\pi \tau +1}\right)\\&\quad+\ln \mathbf{A}\cdot {o}_{\tau }-\ln {\mathbf{s}}_{\pi \tau}, \end{align*}$$

where ${\mathbf{B}}_{\pi \tau}^{\dagger }$ is the normalized transpose of ${\mathbf{B}}_{\pi \tau}$.

### A.2. Planning

In order to treat planning as inference (Botvinick and Toussaint 2012), we must define the free energy to include beliefs about policies. This is

$$F=\boldsymbol{\pi} \cdot \left(\mathbf{F}+\ln \boldsymbol{\pi} \underset{\mathbf{G}}{\underbrace{-\ln {\boldsymbol{\pi}}_0}}\right).$$

If we set the gradient of the free energy to zero, we find

$${\displaystyle \begin{array}{l}{\nabla}_{\boldsymbol{\pi}}F=0\iff \\ {}\boldsymbol{\pi} =\sigma \left(-\mathbf{F}-\mathbf{G}\right)\end{array}}.$$
